# The impact of physical exercise on depression among college students: the mediating role of core self-evaluation and meaning in life

**DOI:** 10.3389/fpsyg.2026.1743318

**Published:** 2026-05-14

**Authors:** Xuesong Ji, Sijie Liu, Zhen Ye, Huajian Shi, Fangyuan Chen

**Affiliations:** 1 The School of Humanities, Arts and Education, Shandong Xiehe University, Jinan, China; 2 Xinhua College of Ningxia University, Yinchuan, China; 3 School of Innovation and Entrepreneurship, Hainan Vocational University of Science and Technology, Haikou, China; 4 Chinese International College, Dhurakij Pundit University, Bangkok, Thailand

**Keywords:** college students, core self-evaluation, depression, meaning in life, physical exercise

## Abstract

**Objective:**

In recent years, increasing attention has been paid to the effectiveness of physical exercise in alleviating depression. This has led to extensive research into its underlying mechanisms. College students are particularly vulnerable to mental health problems due to academic pressure, employment competition, complex interpersonal relationships, and external stress. Drawing on the framework of positive psychological capital, this study examines the role of physical exercise in promoting mental health among college students. It also explores the mechanisms through which such exercise is associated with depression symptoms.

**Methods:**

A total of 1,100 university students were recruited through convenience sampling from universities in Shandong, Ningxia, Hainan, Zhejiang, Guangdong, Guangxi, and Jiangxi. Data were collected using the Physical Activity Level Scale, Self-Rating Depression Scale, Core Self-Evaluation Scale, and Meaning in Life Scale. Additionally, we conducted statistical analyses using SPSS 26.0 and applied Hayes’ PROCESS macro to test the hypothesized chain mediation model and applied bootstrap methods to assess the significance of the mediation paths.

**Results:**

Significant gender differences were identified in physical exercise, core self-evaluation, and meaning in life. Grade-level differences were observed only for meaning in life. Physical exercise was significantly associated with depression, core self-evaluation, and meaning in life. Both core self-evaluation and meaning in life independently mediated the relationship between physical exercise and depression. In addition, a significant chain mediation effect was identified. Specifically, three indirect pathways were significant: (1) Physical exercise → core self-evaluation → depression (indirect effect = −0.047, 95% CI not including 0), accounting for 32.64% of the total effect; (2) Physical exercise → meaning in life → depression (indirect effect = −0.008, 95% CI not including 0), accounting for 5.56% of the total effect; (3) Physical exercise → core self-evaluation → meaning in life → depression (indirect effect = −0.008, 95% CI not including 0), accounting for 25.69% of the total effect.

**Conclusion:**

Physical exercise, depression, core self-evaluation, and meaning in life are significantly associated among college students. Physical exercise is strongly associated with lower levels of depressive symptoms, and is indirectly related to these symptoms through both independent and sequential mediation by core self-evaluation and meaning in life.

## 1 Introduction

With the rapid advancement of China’s economy and technology, amid increasingly fast-paced lifestyles and rising work pressures, a growing number of individuals are experiencing varying degrees of depressive symptoms (Liu J. et al., 2023; Liu Y. et al., 2023). College students are particularly vulnerable to mental health problems as they face substantial academic demands, intense employment competition, complex interpersonal relationships, and heightened psychological stress (Liu J. et al., 2023; Liu Y. et al., 2023; Xiang et al., 2020). This population, therefore, constitutes a distinct group undergoing rapid physical and psychological development. Consequently, they are more susceptible to negative emotions such as depression (Wang et al., 2025). Such symptoms not only impair their academic performance and daily functioning but also place a considerable burden on their families (Boye et al., 2025). Depression is a complex and multifaceted emotional disorder, typically characterized by persistent fatigue, low energy, and low self-worth (Hunter et al., 2025). In severe cases, it may lead to suicidal ideation or behavior. Empirical evidence indicates that between 2013 and 2023, the average prevalence of depression among Chinese college students reached 20.8%, highlighting the seriousness of this issue (Gao et al., 2020). If left unaddressed, depressive symptoms may progress from subclinical states to clinically significant disorders, adversely affecting learning motivation, academic achievement, and engagement in daily activities (Lu et al., 2021). Moreover, depression is associated with an elevated risk of suicide and various physical health complications, including cardiovascular diseases (Liu and Wang, 2024). Therefore, the prevention and alleviation of depression among university students has become a critical concern in both China and the broader academic community.

Against this background, the present study examines the role and mechanisms of physical activity in alleviating depression among college students. Exercise has been widely recognized as an effective non-pharmacological intervention for both the prevention and treatment of depression (Li and Zhang, 2025). It is associated with improved mental health, enhanced quality of life, and reduced stress-induced negative emotions and is linked to lower levels of depressive symptoms (Liu Y. et al., 2024; Liu F. et al., 2024). Regular engagement in such physical activity can elicit positive psychological responses, relieve anxiety and stress, and produce significant antidepressant effects (Lei et al., 2025). However, due to its complex dose–response relationship, exercise is often regarded as a complementary approach in mental health promotion and clinical treatment (Guo and Zhang, 2022). This is partly due to the limited understanding of the mechanisms through which physical exercise is related to depressive symptoms, particularly among college students (Liu Y. et al., 2024; Liu F. et al., 2024). In addition, prior research is often constrained by methodological limitations, such as small sample sizes and narrow research designs, which may limit the robustness and generalizability of findings regarding the antidepressant effects of physical exercise.

In recent years, the effectiveness of physical exercise has received increasing attention, leading to extensive research in alleviating depression, prompting extensive research into the mechanisms through which exercise is associated with depressive symptoms (Zhang et al., 2022). A substantial body of literature suggests that physical activity can effectively regulate mood and behavior, and is positively associated with the overall quality of life (Zhang et al., 2025). Moreover, several studies have identified mediating factors in the relationship between physical exercise and depression among college students (Zhang et al., 2022; Carvalho Silva et al., 2025; Hou et al., 2025). These findings are based on the premise that self-perception and behavior are closely interconnected. Accordingly, depressive symptoms in college students may be shaped by multiple factors, including self-perception and social support (Mao et al., 2024). With the growing emphasis on positive emotions in psychological research, the role of positive psychology in addressing mental health issues, such as anxiety and depression, has attracted growing interest (Shavaisi et al., 2024). As an emerging field, positive psychology focuses on individual strengths and virtues and seeks to promote adaptive functioning in the face of life stressors and psychological challenges (Carr et al., 2024).

Evidence indicates that positive psychological capital plays a crucial role in improving college students’ mental health (Kaiser et al., 2025). In addition, physical exercise has been shown to be positively linked to positive psychological capital by fostering a sense of enjoyment and achievement (Ou et al., 2025). Building on this perspective, the present study examines the impact of physical exercise on mental health among college students through the lens of positive psychological capital and explores its potential underlying mechanisms. Specifically, this study examines university students’ behavior in the context of depression and investigates the sequential relationships among physical exercise, core self-evaluation, and meaning in life. Core self-evaluations reflect individuals’ fundamental appraisals of their own worth and capabilities, including confidence, hope, and optimism about the future, while meaning in life represents a key psychological resource for personal growth and development (Zhao et al., 2024). However, existing research has not adequately examined how physical exercise influences college students’ psychological traits or clarified its relationship with depression. Therefore, this study extends prior work by investigating the effects of physical exercise on depression and related psychological variables using a questionnaire-based approach. The findings may provide valuable insights for promoting mental health, enhancing social engagement, and improving overall wellbeing among university students.

## 2 Literature review and research hypotheses

### 2.1 Physical exercise and depression among college students

Extensive research indicates that physical exercise is significantly associated with both physical and mental health (Hou et al., 2025). Numerous studies show that engagement in physical activity helps individuals release negative emotions, reduce stress, and enhance overall well-being (Zhang, 2025; Jiang and Cao, 2025). Physical exercise generally refers to structured or unstructured bodily activities that contribute to improvements in physical fitness and psychological resilience. Regular participation in such activities strengthens individuals’ capacity to cope with stress and promotes emotional stability (Liu L. et al., 2025; Liu W. et al., 2025; Liu Y. et al., 2025). Empirical evidence further suggests that physical exercise is an effective strategy for preventing depression, anxiety, and related disorders, and may also mitigate the risk of suicidal ideation and behavior (Ou et al., 2025). Accordingly, individuals who engage more frequently in physical exercise tend to report higher levels of self-confidence, self-worth, and life satisfaction. These factors are in turn associated with lower levels of anxiety and depressive symptoms (Zhao et al., 2025).

According to cognitive appraisal theory, individuals’ interpretations and evaluations of external events shape intrinsic motivation, which in turn influences behavior (Zhang and Yusof, 2025). Within this framework, physical exercise can positively affect emotional outcomes by altering cognitive appraisals and enhancing adaptive coping mechanisms. However, the relationship between physical exercise and depression is not strictly linear. Excessive or overly frequent physical activity may have adverse effects, potentially contributing to anxiety, depressive symptoms, or somatization (Liu L. et al., 2025; Liu W. et al., 2025; Liu Y. et al., 2025). Similarly, moderate aerobic exercise has been shown to effectively mitigate depression and anxiety. Nevertheless, excessively intense physical activity may produce mixed emotional outcomes, blurring the distinction between positive and negative effects (Herbert et al., 2020). Overall, while habitual exercise alone may not fully prevent depression, maintaining appropriate levels of activity can significantly alleviate depressive and anxious symptoms, enhance self-esteem, and promote a more positive life orientation (Wei et al., 2024). Building on this body of research, the present study examines the relationship between physical exercise and depression by assessing college students’ levels of physical activity. Based on the above discussion, the following hypothesis is proposed: H1: Physical exercise is negatively associated with depression among college students.

### 2.2 The mediating role of core self-evaluation

Core self-evaluation is a higher-order personality construct covering four interrelated traits: self-esteem, generalized self-efficacy, locus of control, and emotional stability. Together, these dimensions reflect an individual’s fundamental appraisal of their own abilities and self-worth (Hou, 2025). Core self-evaluation is closely associated with mental health both directly, by shaping positive psychological states, and indirectly, by influencing individuals’ self-concepts and cognitive appraisals (Xia et al., 2025). For college students, the sense of self-worth originating from physical exercise is often more accessible than that obtained through academic or occupational achievement (Wei et al., 2024). Participation in physical activity not only enhances physical and psychological well-being but also promotes social interaction and alleviates stress, thereby fostering more positive emotional experiences (Yang et al., 2025). In addition, exercise provides immediate and tangible feedback, such as improvements in physical performance and appearance (Zhu et al., 2024). As a key construct within positive psychology, core self-evaluation has been widely shown to play a significant role in reducing depressive symptoms among college students (Xiang et al., 2025).

From the perspective of cognitive theory, individuals with higher levels of core self-evaluation are more likely to exhibit adaptive cognitive patterns and experience greater psychological resilience (Li, 2025). They tend to interpret challenges more positively and respond to stress with optimism, whereas individuals with lower core self-evaluation are more prone to negative interpretations and maladaptive emotional responses (Liu L. et al., 2025; Liu W. et al., 2025; Liu Y. et al., 2025; Zhuan et al., 2024). Accordingly, core self-evaluation serves as an important indicator of college students’ mental and emotional well-being. Strengthening core self-evaluation may therefore be an effective way for promoting mental health in this population. Based on this rationale, the following hypothesis is proposed: H2: Core self-evaluation mediates the effect of physical exercise on depression among college students.

### 2.3 The mediating role of meaning in life

Meaning in life may serve as another key mediator of depressive symptoms among college students (Zhou and Sun, 2025). Although physical exercise is usually related positively to the overall quality of life, its effects may be constrained, warranting further empirical investigation (Ou et al., 2025). Meaning in life is widely regarded as both a driving force of human existence and a foundation for individuals’ sense of purpose and identity (Vos, 2025). The pursuit of meaning thus serves as both a fundamental motivational force and a critical component of psychological well-being (Song et al., 2025; Tian et al., 2025). Given its strong links to life satisfaction and happiness, meaning in life has become an important focus in mental health research and education (Aldbyani et al., 2025).

Research suggests that physical exercise facilitates the development of meaning in life among college students. Individuals who engage more frequently in physical activity tend to exhibit more positive life attitudes, a stronger sense of purpose, and a deeper appreciation of their own value (Jiang and Bian, 2025). Regular participation in exercise can also promote more adaptive cognitive and emotional patterns (Liang et al., 2020). Furthermore, physical activity may help cultivate a positive life philosophy and worldview, outcomes that are often less readily achieved through other activities. These experiences, in turn, deepen individuals’ reflection on and understanding of life’s meaning (Zhao and Yin, 2024). In addition, engagement in physical exercise can enhance tolerance for setbacks, strengthen psychological resilience, and improve adaptability to social contexts. Based on these considerations, the following hypothesis is proposed: H3: Meaning in life mediates the relationship between physical exercise and depression among college students.

### 2.4 Construction of the chain-based mediating model

Previous research has demonstrated a strong association between core self-evaluation and meaning in life. Specifically, college students with higher levels of core self-evaluation are more likely to pursue meaningful and fulfilling life experiences (Song et al., 2024; Liao et al., 2025). In contrast, those with lower core self-evaluation tend to experience more negative emotions, which may impair concentration, hinder goal setting, and ultimately weaken their sense of purpose and direction in life.

Nevertheless, the interactive mechanisms linking these variables remain unexplored. Existing evidence suggests that individuals with high core self-evaluation typically maintain more positive self-perceptions and greater confidence in their abilities (Song et al., 2024; Liao et al., 2025). Consequently, they are more likely to set challenging goals and demonstrate higher levels of achievement motivation. This, consequently, is positively related to their perceived meaning in life (Zhao et al., 2023). From the perspective of social learning theory, behavioral outcomes influence future behavior through cognitive processes that shape individuals’ perceptions and evaluations (Yin et al., 2025). Within this framework, physical exercise may affect depressive symptoms by influencing key cognitive resources such as core self-evaluation and meaning in life. In addition, research in sports psychology highlights the close interconnection between physical and mental health, suggesting that physical activity is strongly associated not only with improving fitness but also with promoting psychological well-being (Zhang et al., 2022). For college students facing multiple academic and social pressures, developing positive psychological resources, such as self-confidence, optimism, hope, and resilience, is particularly important (Yao et al., 2023). Core self-evaluation and meaning in life can therefore be viewed as sequential cognitive mechanisms through which physical exercise is associated with depression. In summary, previous studies have identified associations among physical exercise, core self-evaluation, meaning in life, and depression. Nevertheless, the underlying pathways linking these variables remain unclear. To address this gap, the present study positions physical exercise as the independent variable, depression as the dependent variable, and core self-evaluation and meaning in life as sequential mediators within a chain mediation framework. Accordingly, the following hypothesis is proposed: H4: Core self-evaluation and meaning in life jointly exert a chain mediation effect on the relationship between physical exercise and depression among college students.

**Figure 1:**
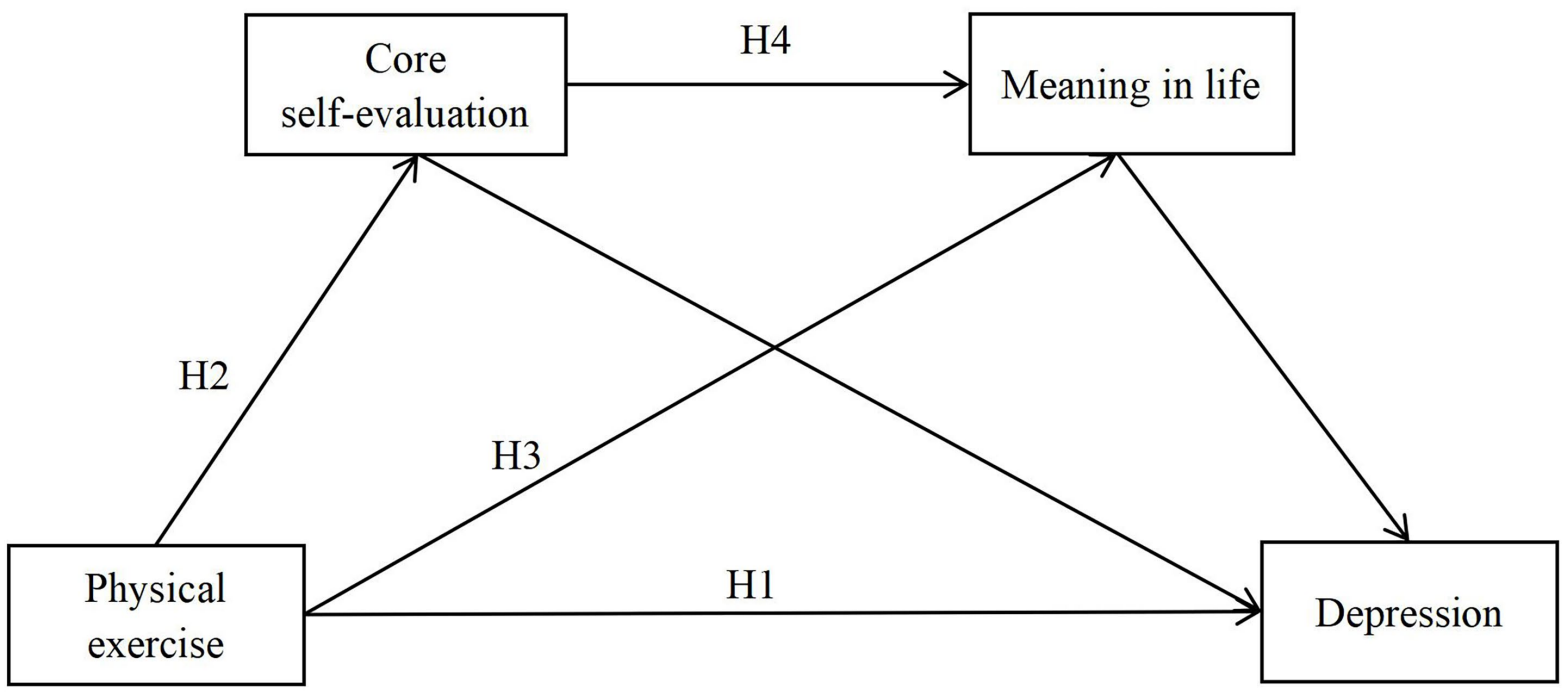
Research hypothesis model.

The hypothesized research model is presented in Figure 1.

## 3 Materials and methods

### 3.1 Participants

An *a priori* sample size calculation was conducted using G*Power 3.1. The parameters were set as follows: effect size (*f* = 0.25), significance level (*α* = 0.05), and statistical power (1-*β* = 0.95), resulting in a required sample size of 210 participants. This study employed a convenience sampling approach to recruit universities across Shandong, Ningxia, Hainan, Zhejiang, Guangdong, Guangxi, and Jiangxi. A total of 1,100 university students were invited to complete an online questionnaire. Among the participating institutions, four were private universities. Participants were further stratified by academic year and major within each institution. Subsequently, simple random sampling was applied within each stratum to select respondents, thereby improving the overall representativeness of the sample.

Clear inclusion and exclusion criteria were established to ensure data quality and generalizability. The inclusion criteria were as follows: (1) full-time enrollment in a university program; (2) absence of physical disabilities; (3) ability to engage in physical activity; and (4) no prior participation in similar studies. Exclusion criteria included failure to meet these requirements, inconsistent or implausible responses, and incomplete questionnaire data. Data were collected using Wenjuanxing, a widely used online survey platform similar to platforms such as Amazon Mechanical Turk. Prior to participation, respondents were informed of the study’s purpose and assured of anonymity and confidentiality. Of the 1,100 questionnaires distributed, 72 were excluded due to invalid or incomplete responses, resulting in a final sample of 1,028 valid questionnaires and a response rate of 93.5%. Among the participants, 467 were male (45.4%), and 561 were female (54.6%). The sample comprised 206 first-year (20.0%), 234 s-year (22.8%), 249 third-year (24.2%), and 339 fourth-year (33.0%) students. The mean age of participants was 19.15 ± 1.05 years.

### 3.2 Measures and procedures for data collection

#### 3.2.1 Physical activity level scale

Physical activity levels were assessed using the revised Physical Activity Rating Scale-3 (Liang, 1994). This scale comprises three dimensions: exercise intensity, exercise duration, and exercise frequency, each represented by a single item. Using a 5-point Likert-type format, exercise intensity and frequency are rated from 1 to 5, while exercise duration is rated from 0 to 4. The overall physical activity score is calculated as the product of exercise intensity, frequency, and duration, yielding a total score from 0 to 100. Scores of 0–19 indicate low activity, 20–42 moderate activity, and 43–100 high activity. In the present study, the scale demonstrated good reliability, with a Cronbach’s alpha coefficient of 0.833.

#### 3.2.2 Self-rating depression scale

Depressive symptoms were measured using the Self-Rating Depression Scale (SDS) developed by Zung (1965). The instrument is widely used in both clinical and research settings due to its brevity and ease of administration. The scale consists of 20 items, with the raw total score obtained by summing all item responses. A standardized score is calculated by multiplying the raw total by 1.25 and rounding to the nearest integer. Higher scores indicate greater severity of depressive symptoms. In this study, the Cronbach’s alpha coefficient was 0.748.

#### 3.2.3 Core self-evaluation scale

Core self-evaluation was measured using the Chinese version of the Core Self-Evaluations Scale (Judge et al., 2003). The scale includes 10 items rated on a 5-point Likert scale ranging from 1 (strongly disagree) to 5 (strongly agree). After reverse scoring the relevant items, a total score was computed, with higher scores indicating higher levels of core self-evaluation. In the present study, the scale demonstrated acceptable reliability, with a Cronbach’s alpha coefficient of 0.734.

#### 3.2.4 Chinese meaning in life questionnaire

Meaning in life was assessed using the Chinese version of the Meaning in Life Questionnaire (MLQ), originally developed by Steger et al. (2006), and adapted by Wang and Dai (2008). The scale includes two dimensions: presence of meaning and search for meaning, both measured on a 7-point Likert scale. Higher scores indicate a stronger sense of meaning in life. Previous validation studies have demonstrated good psychometric properties across different cultural contexts, including American and Japanese student samples. The model showed satisfactory fit indices (χ^2^/df = 2.23, CFI = 0.96, GFI = 0.95, NFI = 0.93, NNFI = 0.95, AGFI = 0.91, RMSEA = 0.069) (Steger et al., 2008). In the present study, the Cronbach’s alpha coefficient was 0.878.

### 3.3 Data processing and analysis

All data analyses were conducted using SPSS 26.0. Following common method bias examination, one-way analysis of variance was applied to examine differences in physical exercise, depression, core self-evaluation, and meaning in life across demographic groups. Subsequently, Pearson’s correlation analysis was performed to assess the bivariate relationships among the four key variables. Finally, the hypothesized chain mediation model was tested using Hayes’ PROCESS macro. The significance of indirect effects was evaluated through the bootstrap method with bias-corrected confidence intervals.

## 4 Results

### 4.1 Common method bias test

To assess common method bias, Harman’s single-factor test was conducted on all measurement items (Harman, 1976). The results indicated that 8 factors had eigenvalues greater than 1, and the first factor accounted for 21.44% of the total variance, which is well below the commonly accepted 40% threshold. These findings suggest that common method bias is unlikely to pose a serious threat to the validity of the results.

### 4.2 Analysis of differences in demographic variables

Independent-samples *t*-tests were conducted to examine gender differences in physical exercise, depression, core self-evaluation, and meaning in life. In addition, multivariate ANOVA was performed to assess differences across grade levels. The results revealed significant gender differences in physical exercise, core self-evaluation, and meaning in life. Male students reported higher scores than female students in these domains. However, no significant gender difference was found for depression. Regarding grade-level differences, no significant variations were observed in physical exercise, depression, or core self-evaluation (see Table 1). Although not statistically significant, meaning in life showed a slight declining trend across grade levels.

**Table 1:** Differences in gender and grade.

Variable	Groups	Statistical value	Physical exercise	Depression	Core self-evaluation	Meaning in life
Gender	Male(467)		13.15 ± 21.45	51.27 ± 9.65	31.76 ± 5.60	4.87 ± 0.99
	Female(561)		9.21 ± 18.08	51.61 ± 8.21	30.30 ± 4.36	4.65 ± 0.97
		*t*	3.198	−0.610	4.722	3.699
		*p*	0.001	0.542	0.000	0.000
Grade	Freshman year(206)		11.69 ± 20.13	50.47 ± 9.53	31.28 ± 4.90	4.88 ± 0.94
	Sophomore(234)		11.58 ± 19.58	52.23 ± 8.93	30.81 ± 5.20	4.64 ± 1.03
	Junior(249)		10.31 ± 18.06	51.75 ± 7.98	30.72 ± 4.61	4.76 ± 0.92
	Senior(339)		10.69 ± 20.92	51.30 ± 9.06	31.06 ± 5.22	4.74 ± 1.02
		*F*	0.282	1.563	0.590	2.311
		*p*	0.838	0.197	0.621	0.075

### 4.3 Descriptive statistical analysis of variables

Pearson correlation analysis examined the relationships among physical exercise, depression, core self-evaluation, and meaning in life. The results are presented in Table 2. Physical exercise was significantly negatively correlated with depression (*r* = −0.320, *p* < 0.001) and significantly positively correlated with both core self-evaluation (*r* = 0.669, *p* < 0.001) and meaning in life (*r* = 0.426, *p* < 0.001). Depression was significantly negatively associated with both core self-evaluation (*r* = −0.368, *p* < 0.001) and meaning in life (*r* = −0.376, *p* < 0.001). In addition, core self-evaluation showed a significant positive correlation with meaning in life (*r* = 0.572, *p* < 0.001). Overall, these results indicate that higher levels of physical activity, core self-evaluation, and meaning in life are associated with lower levels of depression among college students (see Table 2).

**Table 2:** Descriptive statistics and correlation coefficients of the variables.

Variable	M ± SD	Physical exercise	Depression	Core self-evaluation	Meaning in life
Physical exercise	11.00 ± 19.77	1			
Depression	51.45 ± 8.89	−0.320^***^	1		
Core self-evaluation	30.96 ± 5.01	0.669^***^	−0.368^***^	1	
Meaning in life	4.75 ± 0.98	0.426^***^	−0.376^***^	0.572^***^	1

*p < 0.05, **p < 0.01, ***p < 0.001, same below.

Given the significant correlations among variables, potential multicollinearity was further assessed. All predictor variables were standardized, and variance inflation factors were calculated. The results showed that all VIF values ranged from 1.01 to 2.23, all below the commonly accepted threshold (VIF < 5).

### 4.4 Testing the chain mediating effect of core self-evaluation and meaning in life between physical exercise and depression

Given the significant correlations among physical exercise, depression, core self-evaluation, and meaning in life, and lack of multicollinearity, the data met the assumptions required for mediation analysis. All variables were standardized prior to analysis. The PROCESS macro in SPSS was employed to test the hypothesized chain mediation model. Gender and grade were included as control variables. The analysis used 5,000 bootstrap samples to estimate the significance of indirect effects. The settings were as follows: model 6 was specified, with physical exercise as X, core self-evaluation as M1, meaning in life as M2, and depression as Y. In the baseline model without mediators, physical exercise had a significant negative effect on depression (*β* = −0.321, *t* = −10.804, *p* < 0.001), indicating a significant negative association between physical exercise and depression, thus supporting Hypothesis 1.

**Figure 2:**
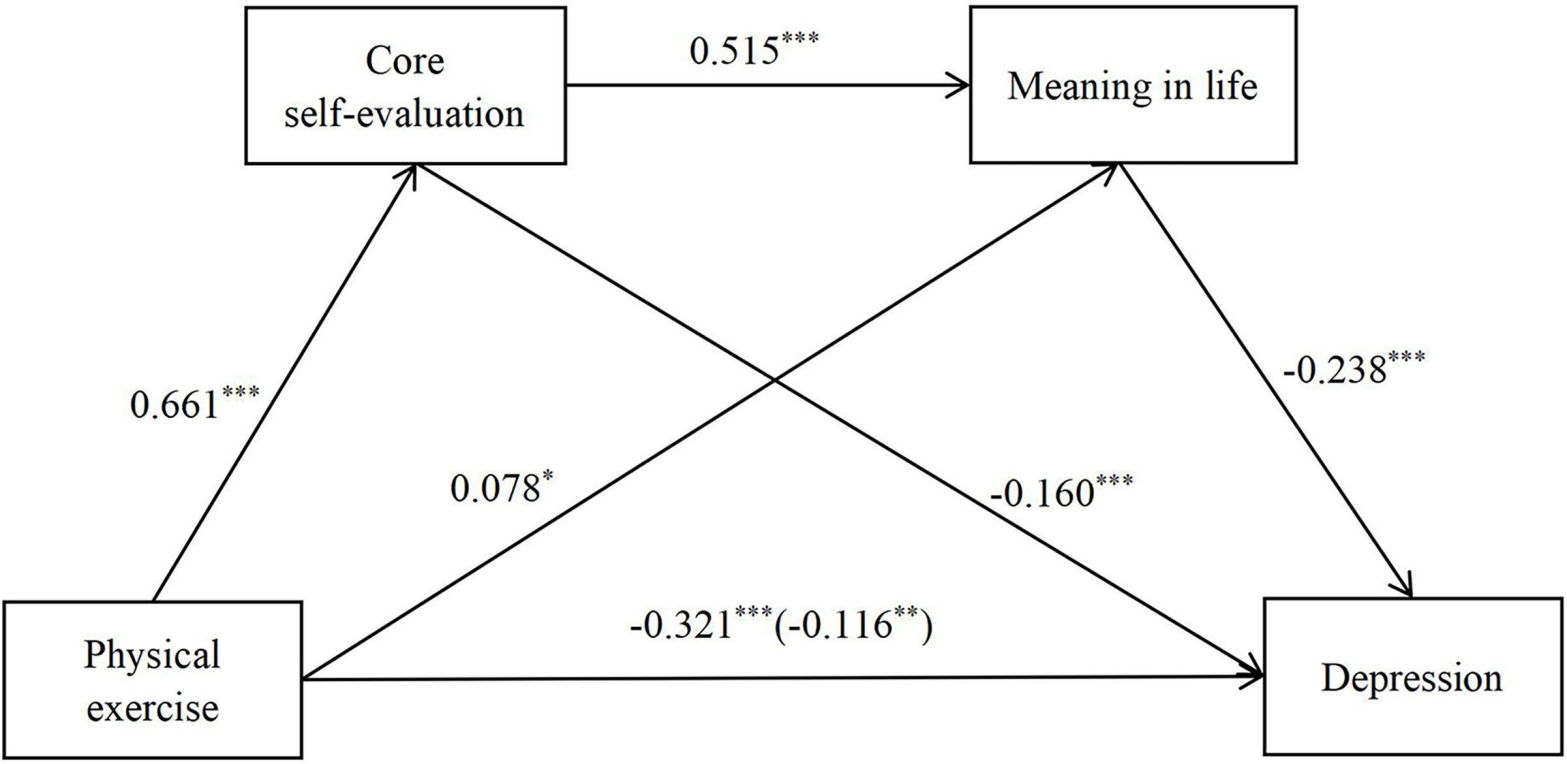
Standardized path of physical exercise and depression in college students.

When core self-evaluation and meaning in life were included as mediators, all hypothesized paths were statistically significant (*p* < 0.01). Specifically, physical exercise was positively associated with core self-evaluation (*β* = 0.661, *t* = 28.487, 95% CI = [0.156, 0.179]), which in turn negatively predicted depression (*β* = −0.160, *t* = −3.805, 95% CI = [−0.431, −0.138]). Physical exercise also positively predicted meaning in life (*β* = 0.078, *t* = 2.269, 95% CI = [0.001, 0.007]), while meaning in life negatively predicted depression (*β* = −0.238, *t* = −6.873, 95% CI = [−2.753, −1.530]). In addition, core self-evaluation significantly and positively predicted meaning in life (*β* = 0.515, *t* = 14.903, 95% CI = [0.088, 0.115]), confirming the sequential (chain) mediation pathways from physical exercise to depression via core self-evaluation and meaning in life (Table 3 and Figure 2).

**Table 3:** Regression analysis of variable relationships.

Variable	Depression		Core self-evaluation		Meaning in life		Depression	
	*β*	*t*	*β*	*t*	*β*	*t*	*β*	*t*
Gender	0.016	0.551	0.079	3.403^**^	0.028	1.101	0.046	1.592
Grade	−0.051	−1.714	0.015	0.629	0.049	1.935	−0.035	−1.232
Physical exercise	−0.321	−10.804^***^	0.661	28.487^***^	0.078	2.269^*^	−0.116	−3.039^**^
Core self-evaluation					0.515	14.903^***^	−0.160	−3.805^***^
Meaning in life							−0.238	−6.873^***^
*R*	0.324		0.674		0.578		0.432	
*R^2^*	0.105		0.454		0.334		0.186	
*F*	40.165		284.103		128.489		46.852	

To further examine the significance of the multiple mediating effects, a bias-corrected nonparametric bootstrap analysis with 5,000 resamples was conducted (see Table 4). The results indicated three significant indirect pathways. First, the indirect effect of physical exercise on depression through core self-evaluation was significant (the indirect effect was −0.047, 95% CI excluding 0), accounting for 32.64% of the total effect. Second, the indirect pathway through meaning in life was also significant (the indirect effect was −0.008, 95% CI excluding 0), accounting for 5.56% of the total effect. Third, the sequential (chain) mediation pathway, physical exercise → core self-evaluation → meaning in life → depression, was significant (the indirect effect was −0.037, 95% CI excluding 0), accounting for 25.69% of the total effect. Comparisons across pathways showed that the indirect effect of core self-evaluation, as well as the chain mediation effect, was substantially stronger than that of meaning in life alone.

**Table 4:** Results of bootstrap-mediated effects analysis.

Impact pathways	Effect	Boot SE	Boot LLCI	Boot ULCI	Effect size ratio
Total effect	−0.144	0.013	−0.170	−0.118	
Direct effect	−0.052	0.017	−0.085	−0.018	36.11%
Total indirect effect	−0.092	0.016	−0.123	−0.058	63.89%
Ind1(PE → CE → DE)	−0.047	0.019	−0.083	−0.009	32.64%
Ind2(PE → LM → DE)	−0.008	0.004	−0.017	−0.002	5.56%
Ind3(PE → CE → LM → DE)	−0.037	0.007	−0.052	−0.023	25.69%
C1(Ind1-Ind2)	−0.038	0.020	−0.076	0.001	
C2(Ind1-Ind3)	−0.009	0.023	−0.054	0.036	
C3(Ind2-Ind3)	0.029	0.009	0.012	0.048	

PE denotes physical exercise; CE denotes core self-evaluation; DE denotes depression; LM denotes meaning in life.

In summary, physical exercise is indirectly associated with depression through both independent mediation (via core self-evaluation and meaning in life) and sequential mediation (via core self-evaluation and meaning in life). These results provide empirical support for Hypotheses 2, 3, and 4.

## 5 Discussion

### 5.1 Sample characteristics

The sample in this study comprised university students aged 17–23 from multiple provinces, including Shandong, Ningxia, Hainan, Zhejiang, Guangdong, Guangxi, and Jiangxi. This developmental stage is a critical transitional period characterized by heightened academic demands, career uncertainty, and identity formation. At the same time, the university environment typically provides convenient access to sports facilities and organized physical activities, which may facilitate regular exercise. The findings revealed significant gender differences in physical exercise, core self-evaluation, and meaning in life, with male students reporting higher levels across these variables. This pattern is consistent with previous research (Wei et al., 2024; Li and Zhang, 2025). One explanation is that many forms of physical activity emphasize competition and performance, contexts in which male students may be more socially encouraged to participate. Engagement in such activities may enhance self-confidence and perceived competence, thereby contributing to a stronger sense of meaning in life.

Furthermore, this study found that perceived meaning in life tends to decline as students progress through their academic years; their perceived sense of meaning in life tends to decline gradually, a pattern consistent with previous research (Liao et al., 2025). One possible explanation is that, upon entering university, students typically hold optimistic expectations about the future and engage more actively in exploring life purpose and personal aspirations. However, as they progress in their studies, increasing pressures related to academics, postgraduate study, and employment may limit such exploration. This growing burden of external expectations and uncertainty can, in turn, weaken students’ sense of meaning in life.

### 5.2 Relationship between physical exercise and depression

The findings of this study, based on both correlation and regression analyses, demonstrate that physical exercise is significantly negatively associated with depressive symptoms among college students. Specifically, higher levels of physical activity are associated with lower depression levels, thereby supporting Hypothesis 1 and aligning with previous research. Physical exercise is associated with mental health through multiple mechanisms. It is linked to higher individuals’ capacity to cope with stress and adversity and promotes positive psychological functioning (Ou et al., 2025; Zhao et al., 2025). As a widely accessible and non-pharmacological intervention, physical exercise has been shown to alleviate depressive symptoms, improve emotional regulation, and foster overall physical and mental well-being. Furthermore, students who engage in regular physical activity are more likely to perceive positive aspects of daily life and maintain a more optimistic outlook (Liu L. et al., 2025; Liu W. et al., 2025; Liu Y. et al., 2025). This positive cognitive-emotional orientation not only enhances subjective well-being but also mitigates vulnerability to depression. Drawing on a large-scale survey of college students, this study provides empirical evidence for the protective role of physical exercise against depressive symptoms (Hou et al., 2025). These findings highlight the importance of promoting physical activity at multiple levels. Educational institutions, families, and society at large should recognize the critical role of physical exercise in mitigating depressive symptoms, particularly through its capacity to enhance positive emotional experiences and strengthen psychological resilience.

### 5.3 Analysis of the mediating role of core self-evaluation

Consistent with Hypothesis 2, the results confirm that core self-evaluation serves as a significant mediator in the relationship between physical exercise and depression among college students, in line with prior empirical findings (Yang et al., 2025). Specifically, engagement in physical exercise is associated with students’ physical and psychological well-being. This in turn strengthens their positive self-perceptions and sense of self-worth (Hou et al., 2025). Physical activity also facilitates positive interpersonal interactions, providing opportunities for social engagement, cooperation, and recognition. These experiences are associated with perceived social support and reinforce individuals’ sense of value and competence. They are thereby associated with higher levels of core self-evaluation (Wei et al., 2024). In addition, physical exercise plays a crucial role in emotional regulation by addressing negative affective states such as anxiety and anger. As a key psychological resource, core self-evaluation strengthens individuals’ capacity for self-regulation and adaptive coping in the face of stress. It fosters a proactive and optimistic orientation toward challenges, thereby reducing susceptibility to depressive symptoms. Therefore, the positive role of physical exercise in promoting college students’ psychological wellbeing should be further recognized by families, educational institutions, and society at large. In guiding students’ participation in physical activities, targeted efforts should help them identify their personal strengths and intrinsic value, thereby enhancing their core self-evaluation. This approach not only mitigates depressive symptoms but also fosters sustainable psychological well-being.

### 5.4 Analysis of the mediating role of meaning in life

Consistent with Hypothesis 3, the findings indicate that meaning in life mediates the relationship between physical exercise and depressive symptoms among college students, in line with prior research (Zhou and Sun, 2025). Specifically, higher levels of physical exercise are associated with a stronger sense of meaning in life and better physical and mental health. These factors, in turn, are linked to lower levels of depressive symptoms (Jiang and Bian, 2025). According to Maslow’s hierarchy of needs, individuals possess an inherent drive toward self-actualization, seeking to discover life’s meaning and realize their potential (Liang et al., 2020). This pursuit reflects not only personal aspirations but also broader social expectations. As a positive psychological resource, meaning in life constitutes a critical internal asset for college students (Zhao et al., 2024). However, when confronted with internal psychological conflicts or external pressures, students may experience a diminished sense of life value, adversely affecting well-being and life satisfaction. Currently, college students in China face multiple challenges, including heavy academic workloads and strained interpersonal relationships, which often generate substantial psychological stress. Exercise provides an effective means of relieving such stress and cultivating a positive mental state. Beyond improving physical health, it enhances self-worth, facilitates social interaction, and reduces anxiety. Through sustained participation in physical activity, students can strengthen emotional regulation, enhance psychological resilience, and increase subjective well-being. All of these improvements contribute to a stronger sense of meaning in life. Therefore, families, educational institutions, and society at large should fully recognize the significant role of physical exercise in promoting college students’ psychological development and life satisfaction. In practice, students should be encouraged to engage in physical activity and to reflect on the meaning and value of life, thereby cultivating a positive sense of purpose and alleviating depressive symptoms.

### 5.5 Chain mediation effect of core self-evaluation and meaning in life

Consistent with Hypothesis 4, the results show that core self-evaluation and meaning in life jointly form a chain-mediated effect on the relationship between physical exercise and depressive symptoms among college students (Guo et al., 2024). This finding suggests that students who engage more frequently in physical exercise tend to demonstrate stronger interpersonal skills, a clearer sense of self-worth, and higher levels of core self-evaluation (Yin et al., 2025). These positive self-perceptions, in turn, facilitate deeper reflection on purpose and quality of life, ultimately reducing depressive symptoms (Zhu et al., 2024). According to social learning theory, behavior is not merely a direct reaction to external stimuli but is shaped by cognitive processes that interpret and shape outcomes. Higher levels of participation in physical exercise are associated with higher core self-evaluation, thereby improving adaptation to campus life. Moreover, engagement in sports and physical activities supports the development of well-rounded personalities and encourages prosocial behaviors, such as cooperation and mutual support. Students with higher core self-evaluation are better able to cope with academic and social stressors, mitigating susceptibility to depression. Meanwhile, both core self-evaluation and meaning in life function as key cognitive mediators that mediate the relationship between physical exercise and mental health outcomes. As the frequency and intensity of exercise increase, students tend to develop more positive perceptions of their physical abilities, experience improved psychological well-being, and experience a stronger sense of meaning in life. Conversely, students who engage in low levels of physical activity often lack effective coping strategies when faced with stress and adversity. This may lead to persistent psychological strain, negative life attitudes, distorted self-evaluation, and, ultimately, elevated levels of depressive symptoms.

In general, engagement in physical exercise contributes to improvements in physical fitness and enhances individuals’ self-confidence, thereby strengthening core self-evaluation. Concurrently, this process facilitates the development of a positive sense of meaning in life, which in turn contributes to the alleviation of depressive symptoms. Pathway comparisons further indicate that both the indirect effect mediated by core self-evaluation and the sequential chain mediation effect are substantially stronger than the effect mediated by meaning in life alone. These findings suggest that, in addition to promoting active participation in physical exercise among students, greater emphasis should be placed on fostering a positive self-concept. Such efforts are of critical importance for the enhancement of both physical and psychological well-being.

## 6 Conclusion, proposal, and prospects

### 6.1 Conclusion

This study identifies significant associations between physical exercise, depressive symptoms, core self-evaluation, and meaning in life among college students. Physical exercise is significantly associated with depressive symptoms and is indirectly related to these symptoms through core self-evaluation, meaning in life, and a sequential (chain) mediation pathway involving both variables.

### 6.2 Implications

The findings offer several practical implications for promoting mental health among college students. First, families, educational institutions, and society at large should collaborate to promote regular physical exercise by integrating it into daily routines and educational settings, thereby ensuring sustained physical and psychological benefits. Second, in addition to providing emotional support, stakeholders should foster students’ core self-evaluation by helping them recognize their intrinsic value and develop a proactive sense of purpose. Third, through coordinated educational and community initiatives, platforms should be established to enable students to express their strengths and realize their potential. Continuous affirmation, constructive feedback, and recognition can enhance students’ confidence, sense of meaning in life, and motivation to maintain healthy lifestyles.

### 6.3 Future directions

This study examined the chain-mediation roles of core self-evaluation and meaning in life in the relationship between physical exercise and depression symptoms among college students. As an early empirical effort to integrate these variables within a unified framework, this study contributes to the literature by clarifying the psychological mechanisms through which physical activity is related to mental health. By validating the proposed chain mediation model, the findings advance understanding of how physical exercise shows a positive relationship with core self-evaluation and the meaning in life, which in turn are linked to lower levels of depressive symptoms. From a practical perspective, it is essential to ensure sustained opportunities for students to engage in physical activity. Meanwhile, stakeholders should also guide them to cultivate positive psychological states during participation, thereby reducing the risk of depression.

## 7 Limitations of the study

This study has several limitations. First, although the sample included students across different genders and academic years, it was based on convenience sampling, which may introduce selection bias and limit the generalizability of the findings to the broader college population of college students. Second, the study relied primarily on self-report measures, which are inherently susceptible to subjective influences, including social desirability bias, cognitive bias, and recall bias. These factors may compromise the accuracy and objectivity of the data, making it difficult to fully capture actual conditions. Third, the research design, wherein all data were drawn from a single source and collected using a uniform method, is vulnerable to common method bias. This may artificially inflate correlations among variables, thereby affecting the validity of the identified relationships and the robustness of the conclusions.

In light of these limitations, several directions for future research are proposed. First, the sampling design should be optimized by expanding the scope and size of the sample and incorporating participants from diverse regions and institutional contexts, thereby enhancing representativeness. Additionally, future studies should adopt a multi-method approach to data collection, integrating multi-source data, objective indicators, and subjective measures to mitigate bias associated with self-reporting. Moreover, common method bias should be rigorously controlled through both procedural design and statistical techniques, in order to more accurately identify relationships among variables and improve the reliability and validity of the findings.

## Data Availability

The raw data supporting the conclusions of this article will be made available by the authors, without undue reservation.
